# Quantification of the Phenomena Affecting Reflective Arterial Photoplethysmography

**DOI:** 10.3390/bioengineering10040460

**Published:** 2023-04-10

**Authors:** Georgios Rovas, Vasiliki Bikia, Nikolaos Stergiopulos

**Affiliations:** Laboratory of Hemodynamics and Cardiovascular Technology, Institute of Bioengineering, Swiss Federal Institute of Technology Lausanne, 1015 Lausanne, Switzerland

**Keywords:** arteries, cardiovascular diseases, health monitoring, hemodynamics, mathematical model, noninvasive, photoplethysmography, optical properties

## Abstract

Photoplethysmography (PPG) is a widely emerging method to assess vascular health in humans. The origins of the signal of reflective PPG on peripheral arteries have not been thoroughly investigated. We aimed to identify and quantify the optical and biomechanical processes that influence the reflective PPG signal. We developed a theoretical model to describe the dependence of reflected light on the pressure, flow rate, and the hemorheological properties of erythrocytes. To verify the theory, we designed a silicone model of a human radial artery, inserted it in a mock circulatory circuit filled with porcine blood, and imposed static and pulsatile flow conditions. We found a positive, linear relationship between the pressure and the PPG and a negative, non-linear relationship, of comparable magnitude, between the flow and the PPG. Additionally, we quantified the effects of the erythrocyte disorientation and aggregation. The theoretical model based on pressure and flow rate yielded more accurate predictions, compared to the model using pressure alone. Our results indicate that the PPG waveform is not a suitable surrogate for intraluminal pressure and that flow rate significantly affects PPG. Further validation of the proposed methodology in vivo could enable the non-invasive estimation of arterial pressure from PPG and increase the accuracy of health-monitoring devices.

## 1. Introduction

Cardiovascular-health monitoring is of vital importance in clinical settings and it is becoming more frequent in daily life, primarily due to the increasing availability of wearable devices. Continuous progress in the field of electronics has resulted in miniaturized biosensors that can be easily embedded in monitoring devices. Many of these sensors utilize photoplethysmography (PPG) to retrieve information about the circulatory system, since PPG has a relatively low cost and is easy to apply. Photoplethysmography was shown to be primarily a measurement of the pulsatile changes in blood volume in perfused tissue and its name is derived exactly from this property. However, this is only partially true for reflective PPG (rPPG) over arterial sites, especially when infrared radiation is used. Understanding the origins of the rPPG signal and its relation to arterial pressure and flow waves is crucial in designing more accurate devices to monitor vascular health. Furthermore, this knowledge could enable the estimation of arterial pressure non-invasively without a trained practitioner; this task remains challenging in clinical practice and in remote monitoring [1].

Many parameters that affect transmissive PPG (tPPG) in thin body parts, such as the fingers and the ear lobe, have been explained in the literature and, therefore, numerous devices based on tPPG are widely adopted in clinical practice [2,3]. Light absorption in the blood increases with increasing blood volume in the illuminated tissue and, hence, the pulsatile (AC) component of PPG is generated [4]. This property is also the reason behind the various features that can be derived from PPG and its time derivatives, some of which have been shown to correlate well with hemodynamic indices and to be accurate predictors of cardiovascular diseases [1,4]. These findings are well accepted in the case of tPPG with visible or infrared illumination, but they cannot directly explain all the phenomena involved in infrared rPPG with higher penetration depths.

Many researchers have investigated the parameters that affect PPG, which can be categorized into individual characteristics, physiology-dependent parameters, and external factors [5]. Individual characteristics that vary within the population, such as age, gender, skin tone and body weight, have been shown to affect the PPG signal and they can usually be corrected with appropriate PPG sensor design and calibration [5,6,7]. The physiology-dependent factors include the respiratory rate, venous pulsations, local temperature, ballistocardiographic artifacts and site of measurement. Techniques such as filtering and machine learning have been used to detect and remove the respiratory-induced variations [8] and the venous pulsations [5,9], while the measurement site has to be selected based on the intended application [10] and the intensity of the ballistocardiographic artifacts [11]. Finally, the design of the sensors can be used in combination with appropriate filtering or machine-learning methods to reduce the influence of external factors, such as motion artifacts [1,12], external light and contact pressure [13]. Although the research on the identification and development of techniques to correct these parameters is essential to the clinical application of PPG-based devices, it is difficult to interpret these findings to elucidate the origins of the PPG signal.

The origins of the rPPG signal are a topic of ongoing scientific debate [9]. It has been suggested that light absorption in the blood determines the amplitude of the rPPG signal, in a manner that is similar to that of the tPPG [14]. Indeed, light absorption constitutes one of the main determinants of the PPG signal. The volume of blood in the illuminated tissue changes periodically due to the pulsatile function of the heart. During the systolic part of the cycle, the arterial pressure increases, along with the arterial diameter. As more blood enters the illuminated region, the absorption of light increases and, hence, the transmitted and reflected light decrease. Recent findings contradicted this theory by proposing the compression of the dermis and the changes in capillary density due to the pulsation of the arteries as the mechanism that forms the PPG signal [15]. However, these results were challenged by subsequent experiments showing that volumetric changes in the arteries and arterioles are responsible for the generation of the PPG signal [16]. Results that contradict both of these mechanisms (blood volume changes and dermis compression), which cannot be explained by changes in the light absorption per se, have also been reported. Blood flowing in transparent rigid conduits generated differences in measured light reflection [17]. Experiments in vivo demonstrated the detection of blood pulsation in teeth and in the patellar bone with light transmission and light reflection, respectively [18,19]. In neither of these two cases was a change in blood volume possible, because the blood was flowing in rigid vessels. Additionally, there are less thoroughly researched phenomena with complex interdependence that affect rPPG. They stem from the interaction of light with biological tissues and the biomechanics of wave propagation in the circulatory system [20]. The reflection of the incident light on the walls of arteries and arterioles can influence the measured reflected light, especially when blue or green visible light is used [16,21]. Moreover, the optical properties of blood, especially of blood in motion, contribute principally to light reflection in the arteries. Many researchers have investigated the optical properties of blood by using a multitude of techniques, some of which are currently used for the estimation of hematological parameters [14,17,22,23,24]. These techniques rely on diluted blood samples or sparse erythrocyte suspensions. Nonetheless, the contribution of the optical properties of flowing blood has not been evaluated and compared to other contributing phenomena in the case of rPPG using whole blood.

The use of rPPG can be more advantageous than tPPG in certain situations. Potential rPPG measurement sites, such as the wrist, provide more comfort for daily use, while tPPG is limited to thin body parts, especially in the case of wearable devices. In thin parts such as the fingers, the pressure and flow waveforms become significantly dampened and they lose potentially valuable information. Therefore, rPPG is more suitable for applications that require the accurate acquisition of the pulse waveform and it could allow more accurate predictions of central hemodynamic indices. Some researchers focused on the association of PPG to pressure and flow waveforms, but their work was mostly limited to finger PPG. Allen and Murray [25] investigated the forward relationship between pressure and tPPG and proposed a model to derive the latter from the former, when both are acquired in the fingers. Moreover, previous research showed that the finger-based tPPG waveform is similar to that of blood pressure in the radial artery and that it can be used as a possible replacement for blood pressure for the detection of hypertension [26]. Despite their similarity, it can be seen that finger PPG and radial artery pressure signals are not identical and no methodology was proposed on how one can be derived from the other. Monte Carlo optical simulations incorporating the pressure–diameter relation of human radial arteries have been used to generate a synthetic PPG waveform based on a pressure waveform [27]. There were, however, significant differences between the experimental and simulated PPG waveforms, especially in the diastolic part, indicating the presence of additional optical phenomena that were not included in the simulation, such as the motion of blood. Lastly, Janjua et al. [28] calculated the correlation between finger PPG and radial artery tonometry in vivo for the prediction of clinical hemodynamical indices. They found a significant correlation only with the augmentation index and the stiffness index, but the evidence was insufficient to reach adequate accuracy to meet the clinical standards. 

The aim of this study was to quantify the optical and biomechanical processes that influence the rPPG signal in vitro. First, we proposed a theoretical method to describe the PPG signal based on the pressure and flow waveforms by combining all the contributing effects. Next, we validated this method in vitro by using an infrared PPG sensor to measure light reflection of an artery model in a mock circulatory system filled with porcine blood. Finally, we calculated the effect of the blood pressure, flow rate and red blood cell (RBC) aggregation on the reflected light by varying the flow conditions and the hemorheological properties of the circulating blood.

## 2. Materials and Methods

### 2.1. Theoretical Approach

The basic principle of rPPG relies on three main processes: (i) a light source illuminates an artery, (ii) the light is absorbed or scattered by the artery and the nearby tissues and (iii) a receiver measures the reflected light. The surrounding tissues remain relatively unaffected by the cardiac cycle and they generate constant intensity of reflected light. By contrast, an artery undergoes significant changes in pressure, diameter and blood flow, which are responsible for the pulsatile component of the PPG signal. The radiant intensity of the light that is reflected from an illuminated artery and, consequently, the irradiance *E* received by a PPG probe, can be expressed as:

(1) $$E=E_{DC}+E_{V}+E_{Q},$$

where $E_{DC}$ is the constant part of irradiance attributed to the illuminated tissues surrounding the artery, $E_{V}$ is the part attributed primarily to the change in volume of the artery and $E_{Q}$ is attributed to blood flow rate changes. Alternatively, $E_{V}$ can be thought of as the flux that is transmitted through the vessel, reflected in surrounding tissues and transmitted back to the receiver, while $E_{Q}$ is the flux that is backscattered in the artery. Although $E_{V}$ and $E_{Q}$ are interdependent, their addition in (1). can be justified, because they both account for minor percentages of the total incident irradiance. Further details on and explanations of the theoretical model can be found in the Supplementary Material.

The measured PPG signal depends on the reflected irradiance and on the operating characteristics of the photodetector. Phototransistors in common collector configuration are a common choice of photodetector and, in this case, the measured PPG signal corresponds to voltage. The response function of the phototransistors is usually given by a power law and we can express the measured PPG voltage as:

(2) $$V_{PPG}=f\left(E\right)=cE^{d},$$

where the function f is defined by the characteristics of the receiver and the circuit and c,d are constants. If d is close to unity, and if the changes in irradiance are small, as those expected in rPPG, (2) can be linearized. This approach could easily be generalized for other types of sensing elements and configurations.

For the blood-volume-related part, we start from light transmission in blood, approximated by a diffusion equation with the assumption of small angle scattering [29]. This approach corresponds to the currently accepted theory that PPG depends on the blood volume in the illuminated tissue [2,3,30] and the varying optical properties of blood due to RBC aggregation and orientation [23]. Consequently, irradiance can be related to pressure as:

(3) $$E_{V}=\frac{E_{0}e^{-\mu_{a}z}}{\cosh\left(z\sqrt{2\mu_{a}{\mu_{s}}^{\prime }}\right)}≅a+kD\left(P\right)≅a^{\prime }+b^{\prime }P$$

where $E_{0}$ is the incident irradiance, $\mu_{a}$ is the absorption coefficient, ${\mu_{s}}^{\prime }$ is the reduced scattering coefficient, z is the distance that light travels in blood, D is the vessel diameter expressed as the pressure–diameter relationship, P is the intraluminal pressure and $a,\;k,\;a^{\prime },\;b^{\prime }$ are constants. In (3), both z and ${\mu_{s}}^{\prime }$ fluctuate during the cardiac cycle, but since their effects on $E_{V}$ combine constructively, they can be expressed as an approximative function of a single variable, for which we chose D(P). Further details on this explanation are included in the Supplementary Material. For small changes in diameter, as in those expected in the radial artery during the cardiac cycle, we can find a linear approximation (third expression) to the second expression of (3). The vessel diameter is related to intraluminal pressure via the elastic properties and the constitutive equation of the arterial wall. The pressure–diameter curve in the human radial artery was found to be approximately linear in the range of 50 to 200 mmHg [31,32]. In human radial arteries, we found a slope equal to 1.1 ± 0.1 × 10^−3^ mm/mmHg by fitting a linear equation (R^2^ = 0.95) on published data [31], which allowed us to derive the last expression without significant errors.

The flow of blood and, specifically, the optical properties of RBCs can be utilized to derive the last term of (1). In detail, RBCs orient along the direction of flow, as blood flows in a conduit. The percentage of aligned RBCs, $p^{*}$, was derived theoretically and measured experimentally [33] and it can be described as follows:

(4) $$p^{*}=m\frac{\sqrt{\dot{\gamma }/\dot{\gamma_{c}}}}{1+\sqrt{\dot{\gamma }/\dot{\gamma_{c}}}},$$

where m is a constant, $\dot{\gamma }$ is the shear rate and $\dot{\gamma_{c}}$ is the critical shear rate. The dimensionless size parameter of RBCs α=πD/λ≫1 in the near infrared; thus, the interaction belongs in the geometric scattering domain. We can approximatively treat RBCs as thin disks. When they are aligned with the direction of flow, they scatter light preferentially towards the lateral direction, which is the direction of measurement in rPPG. When the disks are randomly oriented, they scatter the light in random directions, especially because of their high curvature near the edges of the disk. This behavior has also been shown theoretically [34]. We assume that the percentage of aligned RBCs is proportional to the flow-related irradiance. We define (i) $E_{Q,H}$ as the flow-related irradiance in high shear conditions and (ii) $E_{Q,L}$ as the flow-related irradiance in decelerating and low shear conditions, accounting for disorientation and aggregation. For developed flow in an artery of nearly constant diameter, $E_{Q,H}$ can be expressed as follows:

(5) $$E_{Q,H}=m^{\prime }\frac{\sqrt{Q/Q_{c}}}{1+\sqrt{Q/Q_{c}}},$$

where $m^{\prime }$ is a constant, Q is the average flow rate and $Q_{c}$ is a critical flow rate, which remains constant for each vessel. Equation (5) provides the reflected irradiance based on the average flow rate due to the RBC orientation, while the two constants have to be specified experimentally.

While the RBC orientation occurs relatively quickly compared to the heart period, the RBC disorientation due to the flow deceleration is a slower phenomenon [35,36]. The disorientation of RBCs can be attributed to two aspects: (i) the hydrodynamic conditions and the interactions between flowing RBCs and (ii) the effect of Brownian motion on RBCs [36]. Although the exact mechanisms of disorientation at microscale are outside the scope of the present approach, at macroscale, the disorientation can be described by an exponential decay function. In addition to the orientation, RBC aggregation also influences the optical properties of blood. The RBC rouleaux disperse rapidly when shear stress exceeds a certain threshold value and they start to form when blood is subjected to low shear conditions, thereby creating a reversible aggregation–disaggregation process [37]. Rouleaux formation, as a more ordered state, increases the transmittance of blood by increasing the space that is not occupied by randomly oriented RBCs and, consequently, it increases reflectance. The aggregation of RBCs can also be characterized by an exponential decay function macroscopically [38]. Because RBC orientation and disaggregation are faster-evolving phenomena than their respective counterparts, their effect on measured irradiance can be neglected. As a result, irradiance in low shear conditions $E_{Q,L}$ is given by:

(6) $$E_{Q,L}=E_{Q,H}*\left(n_{d}e^{-\frac{t}{\tau_{d}}}+{n_{a}e}^{-\frac{t}{\tau_{a}}}\right),$$

where $\tau_{d}$, $\tau_{a}$ are the decay constants of RBC disorientation and RBC aggregation, respectively, *t* is the time and $n_{d}$, $n_{a}$ are constants. In practice, only the ratio of $n_{d}$, $n_{a}$ has to be defined as the result of the convolution is normalized with the time integral of the biexponential decay function. The flow-related irradiance $E_{Q}$ depends on the phase of the cardiac cycle; during acceleration or high-shear conditions, it is given by $E_{Q,H}$, while during deceleration or low-shear conditions, it is given by $E_{Q,L}$.

The set of (1)–(6) allows the estimation of the rPPG signal based on the measurement of flow rate and pressure or, equivalently, vessel diameter. Theoretically, it is possible to estimate the calibrated value of PPG by specifying the value of all the constants. However, this would be impractical and redundant, since for most applications, only the uncalibrated value of PPG is required. Thus, it is necessary to calculate only the constants that affect the uncalibrated signal. A summary of the theoretical model and a detailed explanation of the constants are provided as supplementary materials.

### 2.2. Blood Preparation

We purchased whole porcine blood because it was easily available in large quantities and at low cost. In order to avoid blood coagulation, blood was mixed after withdrawal with a 10% solution of EDTA (Merck, Darmstadt, Germany) in PBS, so that a final concentration of 1.8 mg/mL was achieved. Hematocrit was measured with the macro-hematocrit method by centrifuging blood samples for 30 min at 2300 g and hemolysis was estimated with a hemolysis reference palette. In order to inhibit RBC aggregation, we added Poloxamer 188 (Corning Inc., Corning, NY, USA) to a final concentration of 5 mg/mL, which was shown to reduce RBC aggregation [39,40,41] without significantly altering the blood viscosity [42]. Blood was oxygenated with air equal to 4 times the volume of blood, for 15 min at 37 °C on a rollerbank.

### 2.3. Experimental Setup

**Figure 1:**
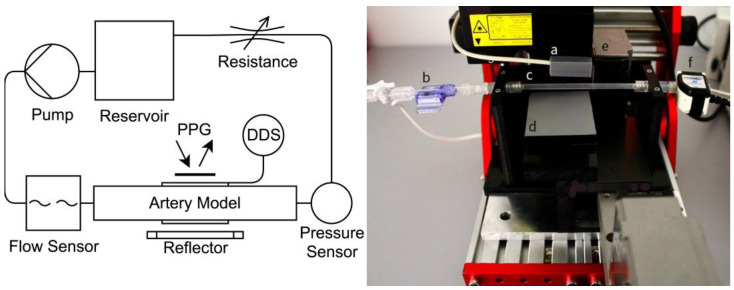
Schematic diagram and photograph of the experimental setup. PPG: reflective PPG sensor, DDS: optical diameter and radial displacement sensor, a: PPG sensor, b: pressure sensor, c: artery model, d: reflector, e: optical diameter/displacement sensor, f: flow sensor.

We created a hydraulic circuit to simulate the blood flow conditions of a radial artery. The circuit was filled with blood and it consisted of a programmable flow pump, a reservoir, a variable resistance and a custom base, on which the artery models, a light reflector and the sensors were inserted (Figure 1). We used stiff tubing for all connections, in order to keep the compliance of the whole circuit at least four orders of magnitude lower than the compliance of the artery model. For the PPG probes, we used wavelength-matched pairs of emitters and phototransistors of varying wavelengths (810 nm, 830 nm and 940 nm). The custom probes provided greater flexibility in comparison to the commercially available probes. The PPG probes were placed over the artery model, while the base allowed precise positioning of the probe in the lateral direction. The probe wavelengths were selected in the first near-infrared window (NIR-I) because NIR-I radiation has sufficient penetration depth to generate adequate signal quality from superficial arteries, while radiation in the visible spectrum is affected more by the increased absorption of the skin and the melanosomes and by the oxygen saturation of blood [43]. Additionally, the probe wavelengths were all above the critical value, below which the optical transmission of blood becomes a non-monotonic function of the size of RBC rouleaux during aggregation. This critical value is 724 nm for human blood, as estimated experimentally and theoretically, while for porcine blood, it is 550 nm, based on the theoretical approximation [23]. The operating curve of the phototransistor was measured by the manufacturer and the exponent was d = 1.3, while the constant c depended on the circuit configuration. We designed an application-specific amplifier with minimal phase delay in the 0.2–10-Hz frequency range to avoid signal distortion in the important part of the frequency spectrum. Depending on the phototransistor, amplification was not always necessary. To model the optical properties of the tissues behind the artery, we used a calibrated card as a reflector, which reflected 18% of the incoming light in the frequency range of the PPG probes.

We performed a series of experiments to isolate the effects of the contributing phenomena. In detail, we performed the following experiments: (i) static, in which the pressure level was changed without blood flow through the artery model; (ii) dynamic with no resistance, in which the blood flow was changed either monotonously or periodically, without significant changes in pressure; (iii) dynamic with resistance, in which both pressure and flow could change; simulating real pulsatile flow conditions; and (iv) dynamic with intermittent flow, in which flow was stopped for 10 s between consecutive pulses.

### 2.4. Artery Models

We designed artery models with semi-transparent, biocompatible silicones (Avantor Inc., Radnor, PA, USA). The models were cylindrical, with constant diameters and thicknesses. Their dimensions and area compliance corresponded to human radial arteries. The refractive index of the models was 1.42, a value close to that of the vascular wall layers [44]. The models were injection-molded and the compliance was measured experimentally by diameter measurement under pressure inflation to verify that it was within the physiological range, between 2.7 and 4 × 10^−3^ mm^2^/mmHg at 100 mmHg [31]. We calculated the slope of the pressure–diameter relationship experimentally for verification purposes via simultaneous pressure and diameter measurements. The constant b was found to be 1.245 ± 0.001 × 10^−3^ mm/mmHg with a linear fit (R^2^ = 0.98). A stiff artery model was also constructed to isolate the effect of flow rate without diameter changes.

### 2.5. Data Acquisition and Processing

The fluid pressure was measured with a calibrated pressure transducer (ADInstruments, Dunedin, New Zealand), the model diameter and radial displacement were measured with an optical micrometer (Keyence Corporation, Osaka, Japan) and the fluid flow was measured with a calibrated tubing flow probe (Transonic Systems Inc., Ithaca, NY, USA). The data from all sensors were recorded simultaneously at a sampling frequency of 1 kHz on a Powerlab (ADInstruments, Dunedin, New Zealand) data acquisition device. The sampling frequency was selected to be sufficiently higher than the relevant frequencies of all recorded signals, which were usually in the 0–40 Hz range. The PPG and flow rate signals were filtered using a low-pass filter with cut-off frequency of 50 Hz to remove the utility frequency and high-frequency noise without affecting relevant frequencies. The PPG values are given in arbitrary units (a.u.) and, according to the most common sign convention, a positive increase in pressure causes a positive increase in PPG or, equivalently, a reduction in reflected light. To calculate the time constants of (6), we fitted a biexponential decay function to the experimental data from the dynamic experiments with intermittent flow using the elastic artery model (see also Supplementary Material). The magnitude-squared coherence function was calculated using Welch’s overlapped averaged periodogram method, with a 2048-point rectangular window and 1024-point overlap.

We performed the statistical analyses and all the aforementioned processing of the recorded data with a custom software programmed in MATLAB (MathWorks, Natick, MA, USA). The means of the disorientation-to-aggregation-constant ratio between control and Poloxamer-infused blood were compared with Welch’s unequal variance t-test. The correlations between measured PPG and the PPG predictions based on pressure, or pressure and flow rate, were evaluated using Pearson’s correlation coefficient (r). We calculated the prediction bias using Bland–Altman analysis, by plotting the difference between predicted and measured values over their average [45]. Linear, as in (3) and non-linear, as in (5) and (6), regressions were performed with the linear and non-linear least-squares method, respectively. The level of statistical significance for all analyses was set to 0.05. All values are reported as mean (SD) or as mean ± SE, accordingly.

## 3. Results

The mean value of the hematocrit was 41% (2), while the percentage of hemolysis remained below 0.8% in the fresh samples and below 1.7% in the used samples. For simplicity and consistency, we present the results from the 830-nm PPG probe without amplification, although probes of varying wavelengths and output power were tested. The other probes yielded similar results. The radial displacement of the artery-model axis did not exceed 0.5% of the diameter change, as measured during the static experiments.

**Figure 2:**
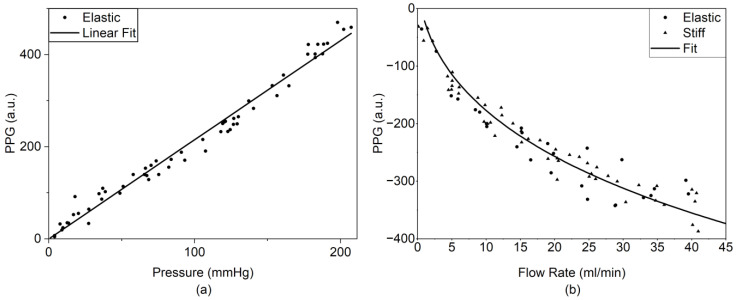
Experimentally measured relationships between pressure, flow rate and PPG at a wavelength of 830 nm. (**a**) The pressure–PPG relationship derived from static experiments without flow and the linear fit on the data (slope 2.15 ± 0.04 a.u./mmHg, R^2^ = 0.98). (**b**) The flow–PPG relationship for elastic and stiff artery models without increase in pressure. The fitted equation corresponds to the degree of alignment of RBCs with flow (5) of the stiff models (m′ = 1613 ± 36 a.u., Q_c_ = 328 ± 21 mL/min, R^2^ = 0.99). The PPG values represent the mean value of PPG over 4 s.

The experimentally measured relationships between the pressure, flow rate and PPG can be seen in Figure 2. The experimental data for pressure were derived from the static pressure–inflation experiments without flow. The linear fit corresponds to the linearized version of (3). The flow rate data were derived from dynamic experiments without resistance. The elastic and stiff models produced similar results, but the elastic model had higher dispersion due to the unavoidable small diameter changes at the high flow rates. Thus, we fitted the function corresponding to (5) based solely on the data from the stiff models, which did not suffer from diameter deviations. Based on the estimated value of the critical flow rate Q_C_ and by assuming Poiseuille flow in the model, we estimated the value of the critical shear rate of the porcine blood to be γ_C_ = 870.0 (55.7) s^−1^.

**Figure 3:**
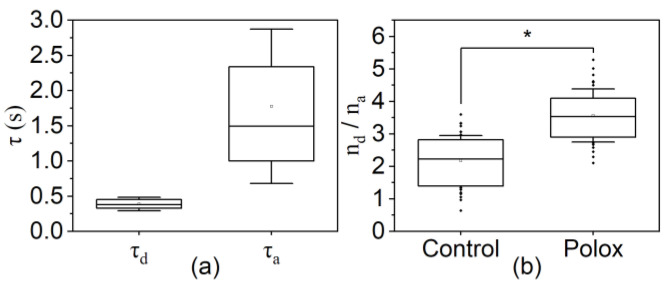
The addition of Poloxamer 188 reduced aggregation. (**a**) The time constants of RBC disorientation τ_d_ and aggregation τ_a_ measured via PPG on whole blood. (**b**) The disorientation-to-aggregation-constant ratio n_d_/n_a_ of whole blood (Control) and of whole blood with Poloxamer 188 (Polox). Box: 25%–75% range, whiskers: mean ± SD, * *p* ≤ 0.001.

We studied the effect of RBC disorientation and aggregation by intermittent-flow experiments. We found that for disorientation τ_d_ = 0.39 (0.09) s and for aggregation τ_a_ = 1.78 (1.10) s. The disorientation-to-aggregation-constant ratio n_d_/n_a_ was 2.2 (0.8) in whole blood and, when we added Poloxamer 188, it was increased to 3.6 (0.8). The Poloxamer reduced the relative contribution of the aggregation by reducing n_a_, resulting in a significant increase in the n_d_/n_a_ ratio (*p* < 0.001). The comparison between the two cases is illustrated in Figure 3.

**Figure 4:**
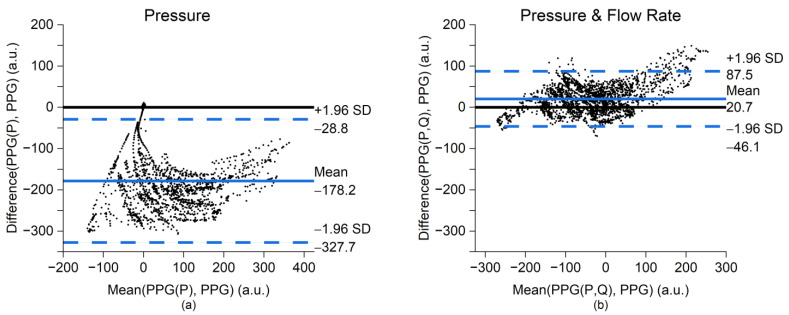
The inclusion of the flow rate dependence of PPG improves prediction accuracy. Bland–Altman plots of the (**a**) predicted instantaneous value of PPG based only on pressure (PPG(P)), and (**b**) based on pressure and flow rate (PPG(P,Q)) versus the measured, instantaneous PPG signal (PPG).

We used the measured values of the constants to predict the PPG signal of the dynamic experiments with resistance, following the aforementioned theoretical approach. First, we used only the pressure signal and we ignored the effect of the flow rate (function PPG(P)), which is equivalent to the hypothesis that PPG is solely a measure of blood volume (Figure 4a). This resulted in a biased prediction (mean = −178.2 a.u., SD = 76.2 a.u.). The Pearson’s correlation coefficient in this case was found to be r = 0.74. Next, we considered both the pressure and the flow rate dependence in the prediction of the PPG (function PPG(P,Q)) (Figure 4b), which improved the prediction accuracy. The prediction was less biased (mean = 20.7 a.u., SD = 34.1 a.u.), while the correlation coefficient increased to r = 0.95.

**Figure 5:**
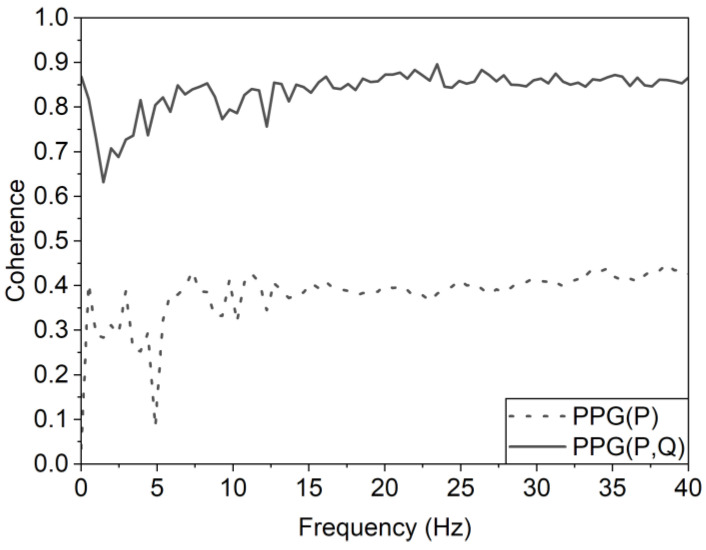
Improved prediction of the PPG signal in the frequency domain. Magnitude-squared coherence of the measured PPG signal and the predicted value of PPG based only on pressure (PPG(P)) or based on pressure and flow rate (PPG(P,Q)).

In addition, the inclusion of both the pressure and the flow rate dependencies improved the accuracy of the prediction in the frequency domain (Figure 5). The mean coherence in the 0–10-Hz frequency range using only pressure as an input variable was equal to 0.32, while when we included the flow rate as an additional input variable, it increased to 0.79. Importantly, the coherence in the latter case remained over 0.6 for all the frequencies in the 0–40-Hz range, which corresponded to the significant part of the frequency spectrum of the measured signals.

**Figure 6:**
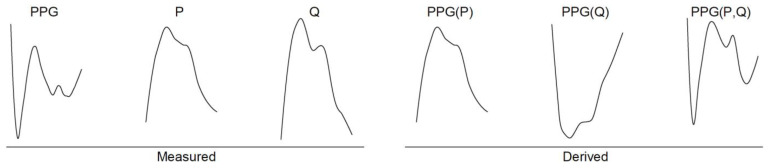
Example of an uncalibrated counter-phase pulse. Measured quantities: PPG (PPG), pressure (P), flow rate (Q). Derived quantities: prediction based only on pressure (PPG(P)), prediction based only on flow rate (PPG(Q)), prediction based on pressure and flow rate (PPG(P,Q)). All waveforms are uncalibrated. The PPG appears to be in counter-phase with pressure and the two waveforms share no similarities. Contrary to the prediction based only on pressure, the prediction based on pressure and flow rate PPG(P,Q) reproduced these counter-phase pulses.

Normally, PPG and pressure are in phase and, given the utilized PPG sign convention, a positive increase in pressure causes a rise in PPG. However, the superposition of the two mechanisms, namely the pressure and flow dependences, generated pulses in which the PPG signal appeared to be in counter-phase with the pressure (Figure 6). Counter-phase pulses occurred in the lower pressure region, with the systolic pressure lower than 100 mmHg, while, for the higher pressure values, the light attenuation clearly surpassed the RBC orientation. In these cases, it was impossible to predict the PPG based solely on the pressure, as the predicted signal was also in counter-phase. By contrast, the combined pressure and flow prediction was in phase with the measured signal. In the latter case, the theoretical prediction accounted for changes in both pressure and flow rate and covered the situations in which the effect of the flow rate on the PPG exceeded that of the pressure, resulting in a negative shift in the PPG despite the rising pressure.

**Figure 7:**
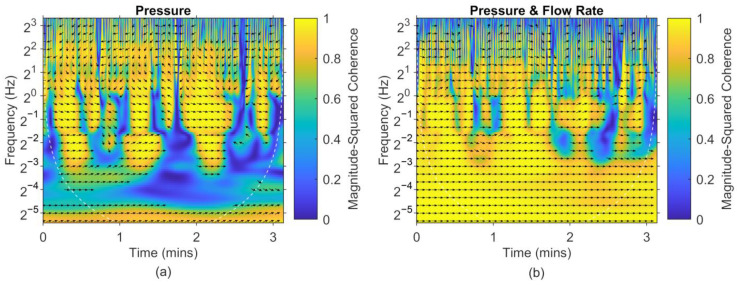
The prediction based on pressure and flow rate improved coherence and phase angle. The magnitude-squared wavelet coherence in the time-frequency plane (**a**) between measured PPG and estimated PPG(P) and (**b**) between measured PPG and estimated PPG(P,Q). The coherence was calculated from a long recording under pulsatile flow with different levels of resistance and mean flow rate. The colormap corresponds to the coherence value. The arrows indicate the phase angle between the two signals, on regions with high (>0.5) coherence. An arrow pointing to the right indicates that the two signals are in phase (0°), an arrow pointing to the left indicates that they are in counter-phase (180°) and intermediate positions indicate the phase delay. The white dashed line signifies the cone of influence.

The combination of the spectral coherence and the phase angle is displayed in the wavelet-coherence graph (Figure 7) in the time-frequency plane [46]. The addition of the flow rate term in the prediction of the PPG signal increased the coherence between the predicted and the measured signal throughout the recording and for most of the frequency spectrum. Crucially, the PPG(P,Q) was in phase or in a small phase delay in relation to the measured PPG for the examined frequency range, regardless of the resistance or mean flow rate value. We did not observe the same behavior in the case of the pressure-based prediction, PPG(P), in which the measured PPG was in counter-phase or in a large phase delay in relation to the PPG(P) in the 1–10-Hz frequency range for long parts of the recording. Consequently, during these parts, the PPG and pressure waveforms were also not in phase.

## 4. Discussion

In this study, we identified and quantified a number of optical and biomechanical phenomena that affect the rPPG signal at peripheral arterial sites, such as the radial artery. Our results suggest that changes in light attenuation due to changes in the vessel diameter or, equivalently, in blood volume, are the major contributors to PPG, accounting for most of its variance, especially under high pressure. Changes in blood flow rate were found to account for the remaining variance, through the alteration of the degree of RBC orientation. Additionally, we showed that RBC disorientation and aggregation both affected the diastolic part of the PPG waveform. The quantification of the aforementioned phenomena allowed us to derive a method to estimate the uncalibrated PPG waveform based on the recorded pressure and flow rate waveforms. To the best of our knowledge, this is the first proposal of this methodology, along with the quantification of the contributing phenomena in the case of the rPPG.

Our experiments showed that the propagating pressure wave is the main cause of the observed PPG signal. The incoming pressure wave distends the vessel, thereby increasing the volume of blood within it, accompanied by a simultaneous change in the optical properties due to RBC disaggregation and orientation. Consequently, the light attenuation within the vessel increases and the amount of reflected light decreases. The pressure or diameter changes were strongly correlated with the changes in PPG, under both static and dynamic flow conditions. This observation was in agreement with the consensus as to the mechanism of operation of tPPG [20]. The causality between the pressure and the PPG was also explored in vivo [26] and it was suggested that arterial blood pressure is the cause of PPG.

In addition to pressure, we also showed that blood flow rate has an important effect on PPG. The alignment of RBCs with the direction of flow and, therefore, the increased blood reflectivity could explain the relationship between flow rate and PPG. The RBC alignment could also explain the PPG pulsation recorded in the patellar bone [19] and in the teeth [18]; these findings contradict the theory that PPG is affected solely by changes in blood volume, because the vessels in the teeth and bones are non-compliant and their volume remains constant. The relation between RBC orientation and shear rate was examined in the past via spin labeling [47] and our experiments produced similar results (Figure 2b). With the assumption of laminar flow and of a parabolic velocity profile, we were able to calculate the value of the critical shear rate for the RBC orientation. Our calculated value agreed with the findings of Bitbol et al. for a human RBC suspension in PBS at 40% hematocrit, γ_C_ = 441 (97) s^−1^. Our assumptions were realistic, since the flow was kept steady during each measurement and the measurement site was far from the entry of the artery model. Similar results regarding the relation of reflected light to the flow rate were reported in another study for rigid conduits and by using a high-power light source and optical fibers instead of a PPG probe [17]. The authors of that study attributed the increase in reflected light with the increasing flow to the migration of RBCs toward the tube axis. While this phenomenon has been verified, at normal values of hematocrit and flow rate, the plasma-rich zone close to the arterial wall does not exceed 4 μm for large-diameter vessels (>100 μm) [48]. In our experiments, this layer would have accounted for less than 0.4% of the total blood volume and, therefore, it would have had a negligible effect on the PPG signal.

The RBC disorientation and aggregation were found to affect the PPG only during the diastolic part of the cycle, in which the flow and the shear rate were close to zero. This caused the RBCs to lose their alignment with the flow and allowed the formation of RBC aggregates. The measurement of the RBC aggregation was possible because we avoided the use of amplification and filtering circuits, which distort the exponential decay of reflected light as aggregates are formed. Our value for the time constant of the RBC aggregation agreed with the reported value τ_a_ = 1.8 (0.2) s for porcine blood, as measured by an optical erythroaggregometer [49]. Our experiments were not designed to measure RBC aggregation accurately and we used varying flow and shear rates, while in aggregometry, a constant shear rate is applied on a thin layer of blood. This could explain the increased variance that occurred in our measurements. To further verify that the decay of the signal was caused by RBC aggregation, we repeated the same measurements with blood treated with Poloxamer 188. This treatment increased the orientation-to-aggregation-constant ratio by 63%, which entailed a reduction in the aggregation of 39%. The latter value, although not directly comparable, agrees with the estimated reduction of RBC aggregation by approximately 40% in the presence of 5 mg/mL Poloxamer 188, as reported for an RBC suspension in Dextran using aggregometry [41]. Regarding the RBC disorientation rate, based on the data and transition times published by Minetti et al. [35], we can calculate the time constant of the disorientation for the decelerating flow at τ_d_ = 0.22 s, which is very close to our estimation. Additionally, other researchers [50] calculated the time constant of orientation in vitro and found a much smaller value, close to τ_o_ = 0.05 s, for high velocities. This justifies our assumption than the orientation occurs much faster and can be neglected; however, it can easily be incorporated in the proposed methodology.

The proposed methodology for the estimation of the PPG waveform based on the pressure and flow rate waveforms produced accurate results. By quantifying the separate effects of the significant mechanisms involved and by utilizing the inherent information embedded in the pressure and flow waveforms, we managed to reduce the bias and the deviation of the prediction and to increase its spectral coherence with the measured signal. The prediction bias in this case is equivalent to the erroneous estimation of the waveform amplitude and, hence, of amplitude-derived indices, such as the amplitude ratio of the systolic-to-diastolic peak. The hypothesis that the rPPG is a measurement of blood volume in larger arteries is challenged by the accuracy of our methodology and by the fact that both pressure and flow influence the PPG at the same order of magnitude. According to the former hypothesis, the estimation of PPG based solely on pressure or blood volume would yield accurate results. As our experiments showed, however, the inclusion of flow rate as a variable is necessary to achieve a precise prediction. The omission of optical effects regulated by the blood flow rate, such as the RBC orientation and aggregation, could also explain the discrepancy between the experimental and simulated PPG waveforms of a recent Monte Carlo computational approach [27]. The researchers simulated the PPG waveform by modeling only the pressure–diameter curve of the radial artery, which is equivalent to the pressure-based PPG prediction presented here. The discrepancy between the experiment and the simulation was larger in the diastolic part of the waveform, where we showed that the effects of the blood flow are the most prominent. The present findings could benefit computational strategies in which the PPG signal has to be estimated based on other hemodynamic parameters. Such strategies are commonly employed for research on the optical sources of PPG [16,27] and for the generation of in silico databases used in non-invasive health monitoring [51]. Crucially, the inverse implementation of our methodology can result in the prediction of the pressure waveform based on the PPG, which is of much higher clinical significance. The pressure waveform contains valuable information that can be extracted by machine-learning algorithms to derive important hemodynamic indices, such as arterial compliance and end-systolic elastance [52,53]. The non-invasive application of these methods currently requires the use of arterial tonometry, which cannot be acquired easily in daily-life settings. The estimation of pressure waveforms with PPG sensors could pave the way for the broad application of similar methods.

Unexpectedly, under certain conditions, we recorded PPG pulses that were in counter-phase with the pressure pulses (Figure 6). As discussed above, the light attenuation had the dominant effect on the PPG waveform; therefore, we expected the PPG to be in phase with the pressure. The counter-phase pulses occurred only when the intraluminal pressure was lower than 100mmHg and, consequently, the volume of the sampled blood was reduced. Similar observations were reported in vivo in the vicinity of the radial artery in another study [15], in which the researchers argued that the volumetric model has to be revised by taking into account the elastic deformations of the dermis. Debating these results, others suggested that counter-phase pulses are caused by motion and ballistocardiographic artifacts [11,16]. In our experiments, neither dermis compression nor motion artifacts were feasible. The counter-phase PPG pulses could be explained solely by the relative contribution of the pressure and flow rate to the measured PPG signal. Due to the low-pressure conditions or the off-center sampling, the blood-volume changes were relatively low and the effect of the flow was comparable to, or even exceed the effect of the pressure. This resulted in a decreasing PPG signal, defined by the negative relationship between the flow rate and the PPG, even though the pressure increased. Therefore, we were able to observe counter-phase PPG waveforms.

To strengthen our understanding of the effect of the contributing mechanisms, we opted to conduct the experiments in vitro, despite the limitations inherent in this approach. This decision can be additionally justified because the simultaneous acquisition of pressure, diameter, flow rate and PPG on the same artery is rather complicated in vivo. Furthermore, porcine blood was used because (i) it is easily available in large quantities and (ii) pigs have similar RBC shape, size and aggregation properties to humans. Another limitation is that we used an artery model instead of a native tissue and we did not interpose vascularized tissues between the model and the PPG probe. Both of these would have added a DC component to the measured signal, which would have exceeded the focus of this study and would have necessitated the use of an amplifier, which would have further distorted the signal, rendering certain measurements impossible. We recognize, however, that arterioles might affect the rPPG in a way that can only be quantified in vivo. Our goal was to construct a model that could be implemented in wearable PPG devices and improve their accuracy. Therefore, we made numerous assumptions to derive a suitable theoretical model at the expense of precision, which was lower than that of a complex theoretical model. Moreover, we used a linear relationship to express the elastic properties of the wall, based on published data for human radial arteries [31,32]. In both the published data and the measurements of our artery model, the coefficient of determination was not improved by using a logarithmic relationship between the pressure and the diameter, a relationship commonly used in most arteries. We also chose to neglect the viscoelasticity of the artery model. We estimated the wall viscosity by minimizing the hysteresis loop of the pressure–diameter relationship and we found the time constant of hysteresis to be within the range of 0.06–0.08 s. This value was low compared to the other time constants and it was neglected. The flow-related irradiance was considered proportional to the percentage of aligned RBCs. We believe this was a reasonable assumption, but it was beyond the scope of this study to provide either theoretical or experimental proof. Finally, oxygenation did not significantly affect our measurements, because oxyhemoglobin and deoxyhemoglobin have similar absorption spectra at the utilized wavelengths [54].

## 5. Conclusions

We conclude that reflective PPG is affected by light attenuation due to changes in blood volume or, equivalently, in blood pressure, by the blood flow rate, as well as by the hemorheological properties of erythrocytes. The quantification of the contributing phenomena allows the accurate derivation of PPG waveforms by measuring the intraluminal pressure and flow rate. Importantly, our findings imply that PPG is not a suitable surrogate for blood pressure and our results provide an alternative explanation for counterphase PPG pulses, which were previously attributed to motion artifacts. We believe that our proposed methodology could broaden the spectrum of biosignals measured non-invasively by health-monitoring devices and increase their accuracy. Our approach can be very beneficial if it is applied inversely, thus enabling the estimation of the pressure waveform from a measured PPG waveform, because the pressure waveform is of much higher biological and clinical significance. Further research is required to determine the extent to which the proposed methodology can be applied in vivo and whether it has a notable, positive impact on healthcare.

## Data Availability

The data presented in this study are available in the article.

## Supplementary Materials

The following supporting information can be downloaded at: https://www.mdpi.com/article/10.3390/bioengineering10040460/s1. Document: Supplementary Material. References [55,56] are cited in the Supplementary Material.
