# Supplementary material for: Quantification of the Phenomena Affecting Reflective Arterial Photoplethysmography
Source: Bioengineering (Basel). 2023 Apr 10;10(4):460. doi: 10.3390/bioengineering10040460 (PMC10136360; doi:10.3390/bioengineering10040460)
Supplement: Supplementary file 1 [file bioengineering-10-00460-s001.zip › bioengineering-2313517-supplementary.pdf]

Supplementary Material

I. THEORETICAL MODEL EQUATION SUMMARY

$$V_{PPG} = f(E) = c\,E^{d} \tag{1}$$

$$E = E_{DC} + E_V + E_Q \tag{2}$$

$$E_V = \frac{E_0 e^{-\mu_a z}}{\cosh\left(z\sqrt{2\mu_a \mu_s{}'}\right)} \cong a + k\,D(P) \cong a' + b'P \tag{3}$$

$$E_Q = \begin{cases} E_{Q,H}\,, & \text{high shear rate / accelerating flow} \\ E_{Q,L}\,, & \text{low shear rate / decelerating flow} \end{cases} \tag{4}$$

$$E_{Q,H} = m' \frac{\sqrt{Q/Q_c}}{1+\sqrt{Q/Q_c}} \tag{5}$$

$$E_{Q,L} = \frac{E_{Q,H} * \left(n_d\, e^{-\frac{t}{\tau_d}} + n_a\, e^{-\frac{t}{\tau_a}}\right)}{\int_0^{t_1}\left(n_d\, e^{-\frac{t}{\tau_d}} + n_a\, e^{-\frac{t}{\tau_a}}\right)dt} = \frac{E_{Q,H} * \left(e^{-\frac{t}{\tau_d}} + \frac{n_a}{n_d} e^{-\frac{t}{\tau_a}}\right)}{\int_0^{t_1}\left(e^{-\frac{t}{\tau_d}} + \frac{n_a}{n_d} e^{-\frac{t}{\tau_a}}\right)dt} \tag{6}$$

II. NOMENCLATURE

a, k, a’, b’	Constants
c, d	Constants
D	Internal arterial diameter expressed as a function of P via the constitutive equation of the arterial wall
E	Reflected irradiance (received by the PPG)
E_0	Incident irradiance
E_{DC}	DC value of reflected irradiance
E_Q	Reflected irradiance attributed to the blood flow rate
$E_{Q,H}$	Reflected irradiance at accelerating/high shear flow conditions
$E_{Q,L}$	Reflected irradiance at decelerating/low shear flow conditions
E_V	Reflected irradiance attributed to the blood volume/pressure
f (E)	Characteristic function of the receiver and the receiver circuit configuration
m', Q_c	Constants
n_d, n_a, τ_d, τ_a	Constants
P	Intraluminal pressure
Q	Blood flow rate
t	Time
t_1	Duration of decelerating/low shear flow conditions
V_{PPG}	Measured voltage of the receiver
z	Depth of tissue / blood layer
μ_a	Absorption coefficient
μ_s’	Reduced scattering coefficient

III. THEORETICAL MODEL EXPLANATION

We divided the theoretical explanation in four illumination cases that also correspond to the in vitro experiments we conducted. The terms introduced in each of these cases refer to Fig. 7. In each case, there are certain assumptions and approximations that are required to derive the measured irradiance. The final, “combined” case best reflects the illumination and hemodynamic conditions that are expected when measuring reflective PPG on a peripheral artery.

Initial Case:

$\mathbf{E_o}$	Incident irradiance
**E**	Measured irradiance
$\mathbf{E_{ri}}$	Partial reflection
$\mathbf{E_{bs}}$	Backscattered irradiance
$\mathbf{E_{ai}}$	Losses due to absorption and scattering
$\mathbf{E_{TR}}$	Tissue reflection

E_{r1} contributes to the constant component of irradiance. The partial reflections E_{r2} and E_{r4} on the arterial wall can be neglected because the refractive index of blood and the arterial wall are similar, resulting in reflectance of less than 0.1%. The tissue reflection E_{TR} is generally higher, due to the larger difference in refractive index between blood and surrounding tissues, e.g. bone, and it is modelled with a partial reflector.

Changes in Pressure:

$\mathbf{E_{ai,1}}$	Losses due to absorption and scattering
$\mathbf{\Delta r}$	Change in the vessel radius

We will show that the measured irradiance can be approximated by a function of a single parameter, the pressure. This is the case because all contributing factors, intuitively, depend on pressure under physiological conditions. The use of the reflector was necessary, as changes only in E_{bs} were not measurable in static experiments. This was expected, because the change in reflectance as calculated by a theoretical approximation [1] was found negligible. As a result, the measured irradiance was primarily affected by the flux that was transmitted through the vessel, was partially reflected on the reflector and was re-transmitted through the vessel.

Under normal conditions, with simultaneous changes in pressure and flow rate, we can distinguish two factors that affect the change in losses and therefore in measured irradiance:

i. The distention of the vessel, Δr, resulting in a change of the depth of the blood layer. This change is reflected in z in (3). With increasing pressure, the vessel distends, the losses increase, and thereby the measured irradiance decreases. The opposite behavior is observed with decreasing pressure.

ii. The change of the reduced scattering coefficient μ_s’ due to the aggregation-disaggregation and orientation-disorientation of red blood cells (RBC). With increasing flow, the pressure increases, the RBCs disaggregate, μ_s’ increases [2], the losses increase, and thereby the measured irradiance decreases. Equivalently, RBC orientation with flow increases μ_s’ and reduces light transmission [3]. The opposite behavior is observed with decreasing flow and pressure.

These two factors both cause a decrease in irradiance with increasing pressure and vice versa. They cannot be easily separated from each other, as an increase in pressure and diameter will unavoidably cause some degree of disaggregation. It could be possible to separate between the two only in vitro, with a slow increase in pressure so as not to disturb the RBC rouleaux, but this is not the case in the cardiovascular system.

Because the effect of both factors is to the same direction, their effects could be combined in a single equation that depends on the diameter or the pressure, third and fourth expressions in (3) respectively. To verify this approach, we examine the case of vertical transmission in the radial artery. We assume a diameter of 3 mm that is subject to a 10% diameter change. We derive the attenuation and scattering coefficients at 830 nm from [4]. Additionally, we expect the reduced scattering coefficient to change up to 4% due to aggregation [4]. The actual change in μ_s’ during a typical heart cycle (0.75 s) will be lower, about 2%, because aggregation does not happen instantaneously. A similar change can also be seen in the results of transmission through RBC suspensions [2], if we consider only the first 0.5 s after flow stop, an amount of time which corresponds to the typical diastolic part of the heart cycle, during which the flow rate is low enough to allow aggregation. However, let us assume a slightly higher value of 5% change in μ_s’ during a heart cycle. Even for these overestimated changes in diameter and μ_s’, (3) is with a good approximation a linear function of the diameter ($R^2 = 0.995$, NRMSE < 1%). If the illumination is not perpendicular to the vessel, or if the probe is placed off-center, the relative change in the optical depth due to the radial expansion of the artery would be even greater. Because of that, the relative contribution of diameter change in the measured irradiance will be further accentuated compared to that of aggregation. To properly model the effects of aggregation and orientation in this case we could follow an approach similar to (4)–(6), because both phenomena start at low shear conditions. The exponential behavior of the transmittance can also be seen in similar in vitro experiments [2]. The result of this approach is a new set of equations that replace (3):

$$E_V = \begin{cases} E_{V,H}\,, & \text{high shear rate / accelerating flow} \\ E_{V,L}\,, & \text{low shear rate / decelerating flow} \end{cases} \tag{7}$$

$$E_{V,H} = \frac{E_0 e^{-\mu_a z}}{\cosh\left(z\sqrt{2\mu_a \mu_s'}\right)} \cong a + k\,D(P) \cong a' + b'P \tag{8}$$

$$E_{V,L} = \frac{E_{V,H} * \left(e^{-\frac{t}{\tau_d}} + \frac{n_a'}{n_d'} e^{-\frac{t}{\tau_a}}\right)}{\int_0^{t_1}\left(e^{-\frac{t}{\tau_d}} + \frac{n_a'}{n_d'} e^{-\frac{t}{\tau_a}}\right) dt} \tag{9}$$

These equations are presented here for completeness. In our experiments, omitting the exponential decay due to aggregation and orientation, or equivalently using (3) instead of (7)–(9), resulted in a maximum relative error of 1%. Therefore, we chose to omit it as it was comparable to the standard deviation of our measurements. Another fact that justifies this approach is that both pressure and diameter decrease gradually until their diastolic value. The almost exponential decrease of pressure during diastole, can sufficiently account for the exponential behavior of transmission due to aggregation and orientation. On the contrary, the blood flow rate decreases rapidly and remains close to zero during diastole, therefore, the effects of aggregation and orientation are more pronounced in the case of blood flow rate changes (vi, third case). The changes in reflectance due to these two phenomena are also treated in the following case.

Summarizing, the effects of diameter change, RBC aggregation and orientation on the light transmission through a vessel reduce transmission with increasing pressure and flow rate, and they can be combined in a single approximative function that depends on one variable. Here, we chose pressure as this variable. From (1), we can calculate the change in measured irradiance when the pressure changes. Under typical conditions, it accounts only for a small percentage of the incident irradiance ($E_1/E_o \in [10^{-7}, 10^{-6}]$). The contribution of the partial reflections E_{ri} can be ignored because $E_{ri} \approx O(E_2\, E_{ri}) \ll O(E_1)$.

Changes in Flow Rate:

$\mathbf{E_{ai,2}}$	Losses due to absorption and scattering
$\mathbf{E_{qi,2}}$	Reflected irradiance due to RBC alignment

The effects of RBC orientation and aggregation cannot be omitted in this case, because they directly affect blood's reflectance, E_{bs}. Hence, it is necessary to use the system of (4)–(6). From (1), we can calculate the change in reflectance due to RBC alignment. Under typical flow rate changes, it accounts only for a small percentage of the incident irradiance ($E_2/E_o \in [10^{-7}, 10^{-6}]$). Moreover, we may neglect $E_{q2,2}$, because $E_{q2,2} \approx O(E_2\, E_1) = O(E_2^2)$. Similarly to the previous case, the partial reflections on the walls can be ignored.

Combined Effects:

$\mathbf{E_{ai,3}}$	Losses due to absorption and scattering
$\mathbf{E_{qi,3}}$	Reflected irradiance due to RBC alignment

In the combined case, the effects of pressure and flow rate changes are superimposed. Although the effects are not technically independent, they both account for minor amounts of the incident irradiance, so that they can be considered practically independent. For example, the reduction in E_3 due to $E_{q1,3}$ will be of the order $O\left(E_1\, E_{q1,3}\right) = O(E_1\, E_2) = O(E_1^2)$ and hence negligible. The same applies to the reduction due to $E_{q2,3}$. Furthermore, the increase in radius will not significantly affect $E_{q1,3}$, because the added layer of thickness Δr will also be filled with aligned RBCs that will reflect the incoming radiation. As a result, the combined effects can be added, as in (2).

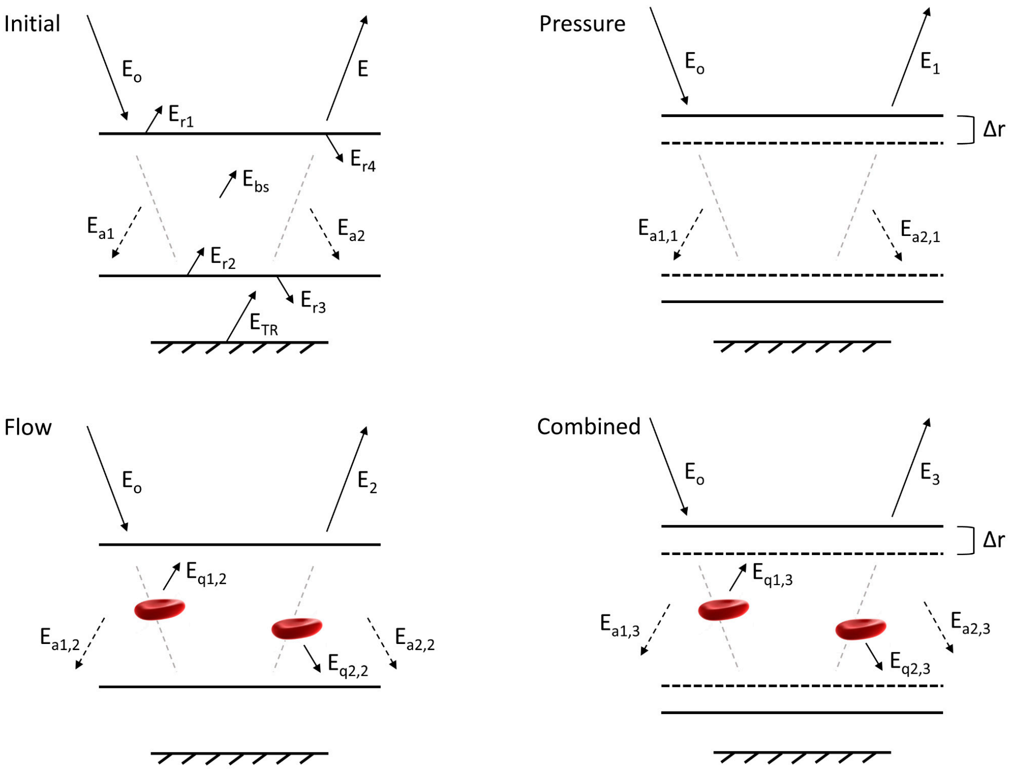

Figure S7. Diagram of the illumination cases of the in vitro setup. Initial: Constant pressure, no flow. Pressure: Static increase in pressure and consequently in diameter, no flow. Flow: Increase in blood flow rate accompanied by red blood cell alignment with the flow direction, no pressure changes. Combined: Increase in pressure and blood flow rate. The symbols are explained in the analysis above. The symbols of the initial case are not repeated in the following cases for simplicity purposes, unless they are significantly affected.

IV. CONSTANT DEFINITION AND DERIVATION

Constant	Definition	PPG type		Derivation
		Calibrated	Uncalibrated	
a	Theoretical value of reflected irradiance of the artery at zero internal diameter	+		Experimentally: Measured value of reflected irradiance at different values of internal diameter under no flow conditions. Linear regression.
k	Slope of the irradiance-internal diameter curve	+	+	
a'	Value of reflected irradiance of the artery at zero-load state	+		Experimentally: Measured value of reflected irradiance at different values of internal pressure under no flow conditions. Linear regression (Fig. 2a)
b'	Slope of the irradiance-internal pressure curve	+	+	
c	Scaling factor of the power law that describes the operating curve of a phototransistor	+	*	Calculated using the characteristics of the phototransistor given by the manufacturer and the corresponding circuit, or else experimentally.
d	Exponent of the power law that describes the operating curve of a phototransistor	+	*	
m'	Scaling factor of the function that describes the dependence of reflected irradiance on blood flow rate[1]	+	+	Experimentally: Measured value of reflected irradiance at different values of blood flow rate. Nonlinear regression (Fig. 2b).
Q_c	Critical value of blood flow rate[1]; if $Q=Q_c$ then $E_{Q,H} = m'/2$	+	+	
n_d	Scaling factor of the exponential decay function due to the disorientation of RBCs.	+	+	Experimentally: Measured value of reflected irradiance after abrupt flow stop. Nonlinear regression (Fig. 8). Only the ratio of the two constants is required. The ratio defines the intensity of reduction in irradiance due to aggregation compared to that caused by disorientation.
n_a	Scaling factor of the exponential decay function due to the aggregation of RBCs.	+	+	
τ_d	Time constant of the exponential decay function due to the disorientation of RBCs.	+	+	Experimentally: Measured value of reflected irradiance after abrupt flow stop. Nonlinear regression (Fig. 8). Because disorientation is more rapid, $\tau_d < \tau_a$.
τ_a	Time constant of the exponential decay function due to the aggregation of RBCs.	+	+	

\+ Required

* Required only if (1) cannot be accurately approximated by a linear function (e.g. when large differences of irradiance are expected or if $d \gg 1$). In this case, the characteristic function of the receiver will distort the measured signal. This distortion will have to be included in the model equations.

[1] These constants were derived by the theoretical equation for the percentage of aligned RBCs as function of the shear rate or blood flow rate.

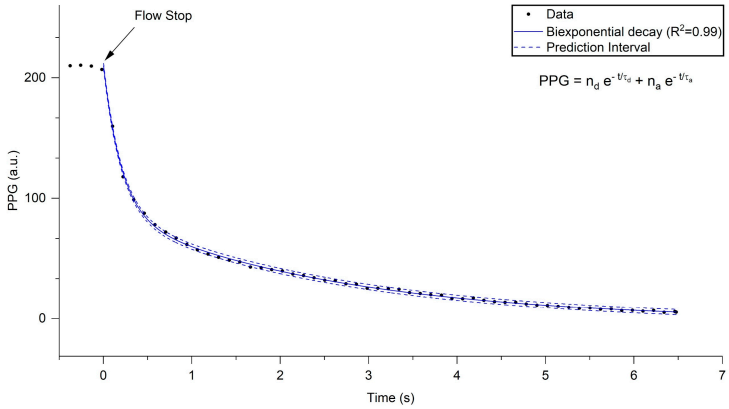

Figure S8. Example of the non-linear regression of the biexponential decay function. Measured value of PPG as a function of time, after abrupt flow stop experiments (points), the fitted function (solid blue line) and the 95% prediction bounds of the fitted function (dashed blue lines). The time that the flow stops is indicated with the arrow. We set the time t=0 s when the flow stops, and the asymptote to zero ($\lim_{t\to\infty} PPG = 0$). There are 4 constants, n_d, n_a, τ_d, τ_a that can be specified by this process. To differentiate between the two time constants, we assume that $\tau_d < \tau_a$, because RBC disorientation is a more rapidly evolving phenomenon, compared to aggregation.

REFERENCES

[1] S. Liang and K. Shimizu, ‘Development of a technique to measure local scattering in turbid media using backscattered light at the surface for noninvasive turbidity evaluation of blood in subcutaneous blood vessels’, *Jpn. J. Appl. Phys.*, vol. 60, no. 2, p. 022002, Jan. 2021, doi: 10.35848/1347-4065/abd36a.

[2] L. D. Shvartsman and I. Fine, ‘Optical transmission of blood: effect of erythrocyte aggregation’, *IEEE Transactions on Biomedical Engineering*, vol. 50, no. 8, pp. 1026–1033, Aug. 2003, doi: 10.1109/TBME.2003.814532.

[3] L.-G. Lindberg and P. A. Oberg, ‘Optical properties of blood in motion’, *OE*, vol. 32, no. 2, pp. 253–257, Feb. 1993, doi: 10.1117/12.60688.

[4] N. Bosschaart, G. J. Edelman, M. C. G. Aalders, T. G. van Leeuwen, and D. J. Faber, ‘A literature review and novel theoretical approach on the optical properties of whole blood’, *Lasers Med Sci*, vol. 29, no. 2, pp. 453–479, Mar. 2014, doi: 10.1007/s10103-013-1446-7.
